# Controlled Release of Highly Hydrophilic Drugs from Novel Poly(Magnesium Acrylate) Matrix Tablets

**DOI:** 10.3390/pharmaceutics12020174

**Published:** 2020-02-19

**Authors:** Rebeca Simancas-Herbada, Ana Fernández-Carballido, Juan Aparicio-Blanco, Karla Slowing, Jorge Rubio-Retama, Enrique López-Cabarcos, Ana-Isabel Torres-Suárez

**Affiliations:** 1 Department of Pharmaceutics and Food Technology, Faculty of Pharmacy, Complutense University of Madrid, 28040 Madrid, Spain; rebecasimancas@gmail.com (R.S.-H.); afernand@ucm.es (A.F.-C.); juan.aparicio.blanco@ucm.es (J.A.-B.); 2 Institute of Industrial Pharmacy, Complutense University of Madrid, 28040 Madrid, Spain; 3 Department of Pharmacology, Pharmacognosy and Botany, Faculty of Pharmacy, Complutense University of Madrid, 28040 Madrid, Spain; karlas@farm.ucm.es; 4 Department of Chemistry in Pharmaceutical Sciences, Faculty of Pharmacy, Complutense University of Madrid, 28040 Madrid, Spain; bjrubio@ucm.es (J.R.-R.); cabarcos@ucm.es (E.L.-C.)

**Keywords:** poly(magnesium acrylate), hydrogel, metformin, matrix tablets, oral controlled release, swelling, erosion studies

## Abstract

The potential of a new poly(magnesium acrylate) hydrogel (PAMgA) as a pharmaceutical excipient for the elaboration of matrix tablets for the extended release of highly hydrophilic drugs was evaluated. The polymer was synthetized with two different crosslinking degrees that were characterized by FTIR and DSC. Their acute oral toxicity was determined in a mouse model, showing no toxicity at doses up to 10 g/kg. Matrix tablets were prepared using metformin hydrochloride as a model drug and the mechanisms involved in drug release (swelling and/or erosion) were investigated using biorrelevant media. This new hydrogel effectively controlled the release of small and highly hydrophilic molecules as metformin, when formulated in matrix tablets for oral administration. The rate of metformin release from PAMgA matrices was mainly controlled by its diffusion through the gel layer (Fickian diffusion). The swelling capacity and the erosion of the matrix tablets influenced the metformin release rate, that was slower at pH 6.8, where polymer swelling is more intensive, than in gastric medium, where matrix erosion is slightly more rapid. The crosslinking degree of the polymer significantly influenced its swelling capacity in acid pH, where swelling is moderate, but not in intestinal fluid, where swelling is more intense.

## 1. Introduction

Metformin HCl (1,1-dimethylbiguanide hydrochloride), belonging to the group of oral hypoglycaemic drugs, is the most used oral treatment in patients with type 2 diabetes mellitus. It exerts its pharmacological action mainly by decreasing both hepatic gluconeogenesis and glycogenolysis. Metformin has low potency and a short biological half-life of approximately 2 h [1]. Consequently, it is used in therapeutics at high-doses and short dose intervals: the usual doses vary between 500 and 1000 mg and they must be administered from 2 to 3 times a day. In immediate-release dosage forms, metformin exhibits an oral bioavailability of 55 ± 16% because, on the one hand, it presents an absorption window limited to the upper segments of the intestinal tract [2]; and, on the other hand, the transporters mediating its absorption are saturable, which makes metformin bioavailability dose-dependent [3]. Moreover, the presence of high concentrations of metformin in the bowel produces gastrointestinal side effects such as diarrhoea, flatulence, vomiting and nausea.

Extended-release tablets are a good alternative to reduce both the gastrointestinal side effects and the frequency of administration thanks to the gradual and prolonged release of the drug [4], and to ultimately improve patient compliance.

Hydrogels are a group of hydrophilic polymer networks that show high capacity to retain in their structure large amounts of water or biological fluids, introducing an elastic modulus and swelling while remaining insoluble and maintaining the shape [5,6,7,8]. This makes them good excipients for extended-release tablets wherein the powdered drug is directly mixed with the polymer and compressed to obtain matrix structures. The rate of drug release from hydrogel matrices depends on the polymer swelling, the drug dissolution and the erosion of the swelled polymer chains [9,10,11].

However, the number of hydrogels used as excipients in the development of new tablet formulations is limited by the need to show adequate mechanical properties, high biocompatibility and a good control of drug release. Indeed, in the case of hydrophobic drugs, the erosion is the main mechanism for drug release, and in the case of biologics such as peptides and proteins, their high molecular size (~400 Da to 30 kDa) limits their diffusion. On the contrary, in the case of small highly water-soluble drugs, their rapid diffusion would make it difficult to achieve an effective control of their release without incorporating hydrophobic polymers [12].

Therefore, the synthesis of new extended release excipients is focusing on developing hydrogels with adequate properties such as compressibility, mechanical strength and modulus, sensitivity to temperature, sensitivity to pH and/or control of drug release. Among the most interesting non-cellulose hydrogels are poly(acrylic acid) derivatives, which are used in topical and oral administration of drugs. These hydrogels show a good biocompatibility and excellent mucoadhesive properties [13,14].

Considering these potential advantages associated with polyacrylic polymers, we synthesized a poly(magnesium acrylate)-PAMgA-hydrogel (fully characterized by Rubio-Retama and López Cabarcos in previous works [5,15]). This hydrogel could be used as an excipient for the development of extended release matrix tablets.

The main aim of this work was to evaluate the potential of this PAMgA hydrogel as pharmaceutical excipient for the elaboration of matrix tablets for the extended release of highly hydrophilic drugs. Metformin was selected as a prototype of small and highly water-soluble drug. The polymer was synthetized with two different crosslinking degrees. In a first step, the thermal and spectroscopic properties of the polymer were determined, and its acute oral toxicity was evaluated in a mouse model. Later, different formulations of metformin matrix tablets were developed, and their drug release profiles were studied using biorelevant media along with the main mechanism involved in drug release (swelling and/or erosion).

## 2. Materials and Methods

### 2.1. Materials

Metformin HCl was purchased from Fagron Iberica (Barcelona, Spain). Sodium taurocholate hydrate, acrylic acid, *N*,*N*,*N*′,*N*′-tetramethylethylenediamine (TEMED), ammonium persulfate (PSA), magnesium hydroxide and pepsin from porcine gastric mucosa were provided from Sigma-Aldrich Spain (Madrid, Spain). Lecithin soy refined was supplied by MP Biomedicals (Illkirch, France). Sodium phosphate monobasic from Acros Organics (Geel, Belgium) and Blanose CMC 7LF were purchased from Ashland Chemical Hispania (Castellón, Spain). All other solvents and reagents (analytical grade) were purchased from Panreac Química (Barcelona, Spain) and utilized as supplied.

### 2.2. Synthesis of the Poly(Magnesium Acrylate): PAMgA

The synthesis of the hydrogel was carried out in two steps: (a) preparation of the monomer (AMgA) and (b) synthesis of the poly (magnesium acrylate) (PAMgA).

The elaboration of the monomer was carried out by a neutralization reaction. An amount of 2 M acrylic acid was added dropwise to a solution of magnesium hydroxide (1 M) in distilled water under constant stirring and controlled temperature (18 °C). When the dispersion changes from off-white to a yellowish transparent solution (24 h), the reaction is concluded. The solution of AMgA is then filtered and concentrated 25% wt. in a rotary evaporator.

The synthesis of PAMgA was carried out by the free radical polymerization method, using ammonium persulfate (PSA) as initiator and TEMED as a catalyst. The hydrogel was synthesized in two different concentrations of PSA: 5 mM (PAMgA 5) and 40 mM (PAMgA 40) that conferred two crosslinking degrees. Polymerization took place at room temperature. The polymers obtained were maintained in distilled water at room temperature for seven days, replacing the water every day. This step was essential to ensure that any rest of unreacted monomer AMgA was removed. Polymer PAMgA was then lyophilized (LIOALFA-6, Telstar, Madrid, Spain), and the powder obtained was sieved and used as excipient to prepare tablets. The two hydrogels obtained were: PAMgA 5, with long segments between linking points in magnesium acrylate monomer chains (AMgA); and PAMgA 40, with short polymer chains between crosslinking points.

### 2.3. Characterization of the Polymer

Differential scanning calorimetry (DSC): A thermal analysis of both polymers, PAMgA 5 and PAMgA 40, was done with a Mettler 820 DSC (Mettler Toledo, Greifensee, Switzerland). The temperature scale was calibrated using the melting temperatures of indium and zinc. The rate of dry nitrogen flow and rate of heating were 80 mL/min and 10 °C/min, respectively. Samples (4–5 mg) were double scanned with a heating range from −90 °C up to 250 °C. An empty aluminium pan was used as reference.

Fourier transform infrared spectra (FTIR): Spectra were recorded on a Nicolet IR200 spectrophotometer (Thermo Fisher Scientific, Waltham, MA, USA) within a range of wave number 400–4000 cm^−1^ at room temperature. Potassium bromide (KBr) pellet method was employed.

### 2.4. In Vivo Oral Biocompatibility Study of PAMgA

High doses were used to evaluate the acute toxicity of PAMgA 5 and PAMgA 40. Animal procedures were approved by the Ethics Committee for Animal Experimentation of Madrid (PROEX 148/17, 3 of November of 2017, Madrid, Spain) and conducted according to European Community Council Directive 010/63/UE. The study was performed with adult male Swiss mice (30 ± 2 g) which were divided in six different groups that received PAMgA polymer at different doses. Mice were kept in standardized conditions of temperature 22 ± 3 °C, 12 h light on/off cycle, feeding and water ad libitum. The polymer was administered at the solid state, mixed at different proportions with maintenance food and forming pellets with a total weight of 2 g. To ensure the ingestion of the polymer, mice were deprived of food 24 h prior to the administration and pellet consumption was allowed for a maximum time interval of 4 h. To determine the exact amount of hydrogel ingested by each mouse, the difference between the initial weight of the pellets (2 g) and the final weight remaining after ingestion was calculated. To unify the dose, when the weight of the ingested pellet deviates from ± 20% of the reference values (Table 1), the animal was rejected.

General behaviour of mice and symptoms such as changes in mobility, vomiting, restlessness, squeaks and diarrhoea were constantly overseen during the first hour after ingestion, and then after 4, 8, 24, 48 and 72 h, as previously reported [16]. The response of animals to oral administration was assessed on a 0–2 scale as follows. Zero correlates with a no response, or normal response; 1 refers to only one episode or a light response, and 2 is more than one event or a severe response to oral administration. Mice were observed and weighed after 24, 48 and 72 h following the oral administration to detect any signs of toxicity and mortality. At the end of the experiment, the animals were sacrificed and dissected for the observation of the organs.

### 2.5. Preparation of Tablets

Tablets were prepared using both types of powdered PAMgA: PAMgA 5 and PAMgA 40, as excipients for matrix tablets. The active ingredient, metformin HCl, was used as the supplied powder or previously granulated by wet granulation. For the granulation process, a 5% aqueous solution of NaCMC (Blanose CMC 7LF) was used as binder; the wet mass was forced through a 1 mm aperture size sieve; the granules were then dried at 35 °C for 24 h and subsequently sieved through a 1 mm sieve. Metformin, powder or granules, was blended with the polymer in a 50% *w*/*w* ratio. Tablets were obtained by direct compression with a single-punch eccentric tableting machine (J. Bonals 30B, Barcelona, Spain) using 14 mm biconcave oval punches. Table 2 shows the composition of the four different batches of tablets prepared.

### 2.6. In Vitro Drug Release

To determine the release profile of metformin from tablets, biorelevant media were used: Simulated Fasted Gastric State (FaSSGF) pH 1.2 and Simulated Fasted State Conditions in the Small Intestine (FaSSIF) at pH 6.8. These media resemble physiological conditions of the gastrointestinal track [17] as they incorporate substances that may modify the dissolution rate of the drug, such as pepsin, lecithin or sodium taurocholate [18]. The composition of the biorelevant media FaSSGF and FaSSIF is shown in Table 3.

Dissolution tests were carried out using USP dissolution apparatus type II (708-DS, Agilent Technologies, USA), regulated to 100 rpm and 37 ± 0.1 °C. With the purpose of determining the way in which metformin incorporation and the different degree of crosslinking influences drug release from the matrix tablets, two distinct biorelevant media (namely, FaSSGF or FaSSIF (1000 mL)) were used as dissolution medium. Aliquots of 5 mL were withdrawn at predetermined time intervals (15, 30 and 45 min, 1, 2, 3, 4, 5, 6, 7, 8, 10 and 24 h) and filtered through 0.45 µm membrane filter (Millex-LCR, Millipore). Drug concentration was determined by spectrophotometry UV at 232 nm (DU-7 Spectrophotometer UV-Vis, Beckman Coulter, USA). Experiments were performed in triplicate for each formulation and results are expressed as percentage of drug dissolved (mean ± SD). Dissolution data were fitted to the exponential equation used to evaluate the behaviour of controlled release pharmaceutical forms—Korsmeyer Peppas release model (Equation (1)) [19].

This equation is used for polymeric systems where the release mechanism is not well known, or more than one type of release behaviour is involved. Mt/M∞ is the fraction of drug released at time t, k is the kinetic constant, which depends on the geometrical properties of the tablet, and n is the release exponent for the drug.

(1) $$\frac{Mt}{M\infty }=k\;t^{n}$$

For a cylindrical matrix, *n* values < 0.45 indicate Fickian release mechanism (diffusional controlled release); 0.45 < *n* < 0.89 indicate an anomalous release (non-Fickian) and *n* > 0.89 indicate Case II transport kinetics (relaxation-controlled delivery). Only the data corresponding to *Mt*/*M*∞ values below 0.60 were used to calculate the value of the release exponent.

The dissolution profiles of tablet formulations were compared by the difference factor (f1) and similarity factor (f2) using the Equations (2) and (3), respectively.

(2) $$f1=\frac{\sum_{t=1}^{n}\left|Rt-Tt\right|}{\sum_{t=1}^{n}Rt}\cdot 100$$

(3) $$f2=50\cdot \log\left\{{\left[1+\frac{1}{n}\cdot \overset{n}{\underset{t=1}{\sum }}{\left(Rt-Tt\right)}^{2}\right]}^{-0.5}\cdot 100\right\}$$

where *n* is the number of time points, and *R_t_* and *T_t_* are the percentage of drug released at time t of each of the pairs of metformin tablets compared [20,21,22]. Only one measurement was considered after 85% of the drug had been released. When the *f*_1_-value is between 0 and 15%, the curves were considered overlapping and with *f*_2_-values between 50 and 100 were considered similar. At last, dissolution data at pH 6.8 of the best formulation was analyzed in order to determine if it meets the specifications listed in the USP monograph of Metformin hydrochloride extended release tablets.

### 2.7. Swelling and Matrix Erosion Behaviour

The dissolution apparatus USP type II at 100 rpm and 37 ± 0.1 °C was used. In this assay, 250 mL of biorelevant media (FaSSGF or FaSSIF) was used [18]. The matrix tablets (F3 and F4) were weighed and placed in the dissolution vessels containing the medium. The tablets were withdrawn at different time intervals: 1, 4 and 8 h, slightly blotted with a tissue paper to remove excess of medium and re-weighed to calculate swelling capacity. Then tablets were dried in a hot-air oven at 50 °C until constant weight to calculate the erosion percentage. Swelling capacity (SW) and matrix erosion (E) were estimated using Equations (4) and (5) respectively, where W_o_ is the initial weight of the dry matrices; W_f_ is the final weight after drying; W_i_ is weight of the swollen matrices after time t; Mt is the amount of drug released at time t; M∞ is the maximum amount of drug released and W_drug_ is the initial weight of drug, as previously described [23].

Both the matrix erosion data and the swelling capacity of the tablets were expressed as mean ± SD of three determinations.

(4) $$SW\;\left(\%\right)=\frac{Wi-Wo}{Wo}\times 100$$

(5) $$E\;\left(\%\right)=\frac{Wo-Wf-Wdrug\;\left(1-\frac{Mt}{M\infty }\right)}{Wo}\times 100$$

At the same time intervals, morphological studies of the tablets were carried out following the procedure described elsewhere [24].

These studies are useful to determine the changes that occur in the matrix tablets during dissolution assays. For these experiments the tablets of batches F3 and F4 were withdrawn from the dissolution vessels after 1, 4 and 8 h. The tablets were blotted to remove excess of medium and sliced in half. Images of the tablets were taken using a camera NIKON COOLPIX S9100 (Nikon España, Barcelona, Spain), and subsequently analysed with IMAGE PRO PLUS 6.0 software.

## 3. Results

### 3.1. Characterization of the Polymer

The yield obtained in the synthesis of the polymer was high for both crosslinking degrees, but higher for PAMgA 40 (85.0%) than for PAMgA 5 (71.2%). No significant differences in the appearance of the polymers were observed, obtaining, in both cases, an off-white powder after freeze-drying.

**Figure 1:**
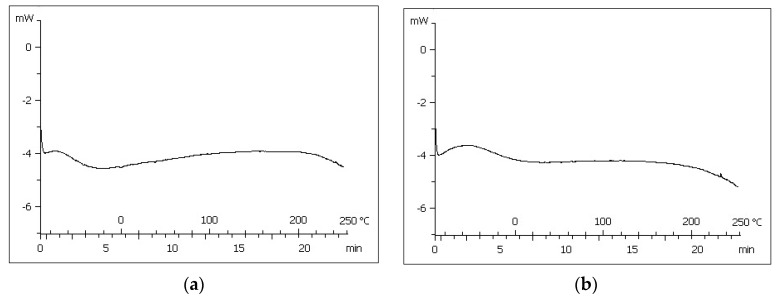
Second cycle DSC thermogram of (**a**) PAMgA 5 and (**b**) PAMgA 40.

DSC curves of both PAMgA 5 and PAMgA 40 are shown in Figure 1. Both polymers were double scanned with temperatures between −90 and 250 °C. The first heating showed a wide endothermic peak around 100–120 °C, which disappeared in the second heating, so this peak corresponds to the loss of moisture present in the polymer. Glass transition or melting events were not identified in this temperature range.

**Figure 2:**
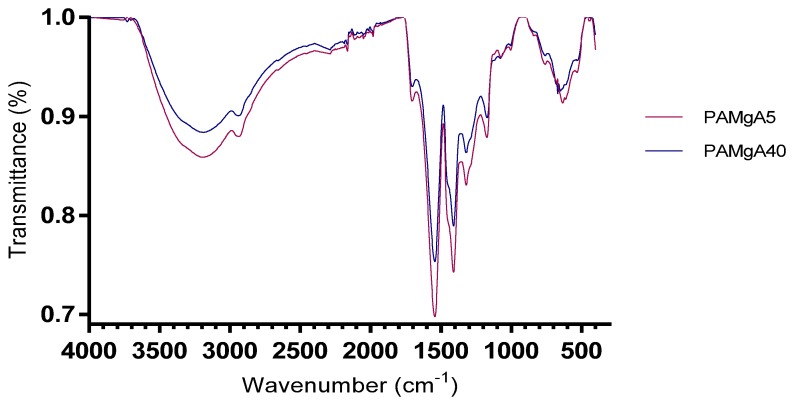
FT-IR spectrum of PAMgA 5 and PAMgA 40.

FT-IR spectra of both polymers, PAMgA 5 and PAMgA 40, were recorded using KBr pellet method (Figure 2). The broad peak in the region 3550–2500 cm^−1^ is attributed to the –OH stretching vibrations of the carboxylate groups. The C=O groups of the carboxylate groups are also associated with two specific bands in the range 1610–1400 cm^−1^. These bands appear as an asymmetric stretching vibration between 1610–1550 cm^−1^ and a symmetric stretching vibration peak of the –COO– in the 1450–1400 cm^−1^ range.

### 3.2. In Vivo Oral Biocompatibility Study

The acute toxicity of both PAMgA hydrogels was evaluated in mice after oral administration of different doses by using pellets that were made from food mixed with each hydrogel. No signs of toxicity were observed at all tested doses, and even at the highest dose range studied (10 g/kg) both hydrogels were well tolerated (Table 4). All animals survived at the end of the experiment without any significant symptomatology. However, at the highest dose tested, some of the animals experienced diarrhoea after 24 h of administration. There were no changes in body weight and food consumption in any of the animals, and the visual analysis of the liver, spleen, lungs, kidney, stomach and intestine of the animals after euthanasia did not reveal any changes in their morphology.

### 3.3. Characterization of the Tablets

Table 5 shows the characteristics of the tablets prepared from PAMgA 5 and PAMgA 40 polymers. The drug was incorporated in a 50% *w*/*w* to the matrix tablet, giving rise to oval tablets with suitable dimensions for oral administration and an approximate weight of 1 g. The differences in hardness among formulations were due to the different way of incorporation of metformin HCl, since in formulations F3 and F4 the drug was incorporated as granules with NaCMC as binder, which facilitated the direct compression of the blend and improved the mechanical resistance of the tablets. The direct mix of the drug powder and the polymer was difficult to compress and tablets did not take hardness and tended to capping.

### 3.4. In Vitro Drug Release

**Figure 3:**
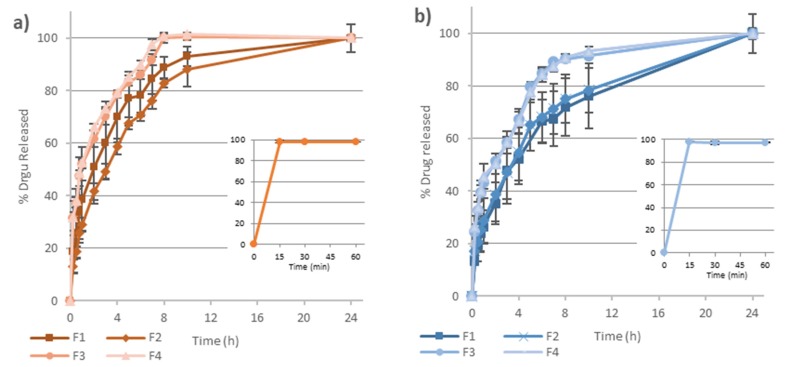
Accumulative percentage of dissolved metformin HCl for formulations F1–F4 matrix tablets (large graphics) and drug powder (small graphics) in (**a**) FaSSGF at pH 1.2 and (**b**) FaSSIF at pH 6.8 (n = 3).

The accumulative percentage of metformin released versus time from the four batches of matrix tablets prepared is shown in Figure 3. In the dissolution assay of the free drug, the dose was immediately dissolved (within the first 15 min) at both pHs studied (Figure 3-small graphics).

On the contrary, when formulated into PAMgA matrix tablets, metformin release was prolonged for at least 6–8 h. On the one hand, in FaSSGF not less than 85% of the drug was released from both F3 and F4 in 6 h and for F1 and F2 this time increased up to 8 and 9 h respectively. On the other hand, in FaSSIF, formulations F3 and F4 released 85% of metformin HCl at 7 h, whereas F1 and F2 reached this percentage after more than 10 h.

To calculate the difference factor and the similarity factor, the number of data points was fixed (n = 5) for all formulations. To determine if the polymer crosslinking affects the drug release, F1–F2 and F3–F4 were compared, in both media of pH 1.2 and 6.8. Furthermore, to determine if the way in which metformin HCl is incorporated in the matrix affects the drug release, formulations F1–F3 and F2–F4 were also compared. Table 6 shows the results obtained for all these comparisons.

In FaSSGF, F1–F2 presented *f*_1_ and *f*_2_-values of 17.2 and 51.0 respectively, which do not allow to determine in a clear way the similarity of both dissolution profiles. For F3–F4 comparison, *f*_1_ and *f*_2_-values of 2.8 and 81.4 were obtained indicating similarity between both dissolution profiles. When F1 and F3 formulations were compared, *f*_1_ and *f*_2_ values (15.8 and 49.1) are in the limit of the non-similarity; and when F2–F4 formulations were compared, the value of *f*_1_ was clearly greater than 15 and the value *f*_2_ smaller than 50, which indicates no similarity.

In FaSSIF, results were clearer than those obtained in acid pH, with values of *f*_1_ and *f*_2_ for formulations F1–F2 of 4.2 and 80.5 respectively, and for F3–F4 of 0.4 and 92.7, which confirm the similarity of the compared release profiles. Conversely, both the comparison of F1–F3 (with *f*_1_ and *f*_2_-values of 39.8 and 26.8, respectively) and of F2–F4 (with *f*_1_ and *f*_2_-values of 42.5 and 23.8, respectively) evidenced the non-similarity between their metformin release profiles.

When metformin release data were fitted to Korsmeyer-Peppas equation, the correlation coefficient (r^2^), release rate constant (k), and release exponent (n) shown in Table 7 were obtained. All the release profiles fitted well the Korsmeyer-Peppas model, with correlation coefficients (r^2^) above 0.98 in all cases both in FaSSGF and FaSSIF. The value of the release constant, k, was higher for F3 and F4 formulations, especially in FaSSGF, suggesting a faster drug release from these matrices. For F1 and F2 the diffusional exponent (n) was in the range of 0.45 < n < 0.89, indicating that the release of the drug from the tablets was a combination of more than one mechanism, diffusion of the drug through the hydrated gel layer and matrix erosion. In the case of F3 and F4, the n-value was below 0.45, indicating that the release mechanism was mainly controlled by diffusion of the drug from the gel layer.

### 3.5. Swelling and Erosion Behaviour

**Figure 4:**
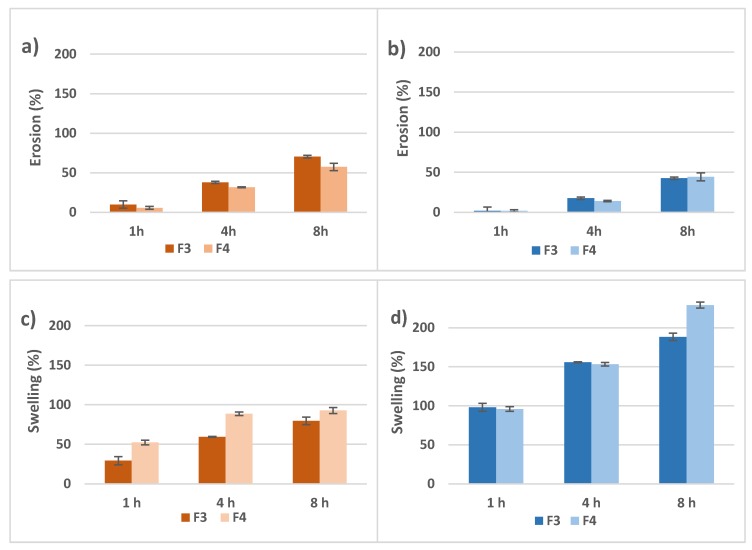
Accumulative erosion percentages for F3 and F4 in (**a**) FaSSGF and (**b**) FaSSIF and accumulative swelling percentages for F3 and F4 in (**c**) FaSSGF and (**d**) FaSSIF.

Figure 4 shows swelling and erosion data for both F3 and F4 tablet formulations in both pHs evaluated. The erosion percentage for formulations F3 and F4 was 9.9 ± 4.7% and 5.7 ± 1.9% respectively, in the first hour of the experiment in FaSSGF (Figure 4a). These percentages increased up to 70.4 ± 1.6% and 57.4 ± 4.6% at the end of the experiment (8 h). In FaSSIF, the erosion percentage was negligible in the first hour of the experiment for both F3 and F4 but at the end of the experiment the erosion of F3 and F4 matrix tablets reached 42.5 ± 0.9% and 44.2 ± 0.2%, respectively (Figure 4b).

Regarding swelling, after 1 h in FaSSGF, the swelling percentage of F3 and F4 were 29.4 ± 5.2% and 52.2 ± 2.9% respectively, and after 8 h, these percentages increased up to 79.4 ± 4.7% and 92.6 ± 3.8%. These values were significantly lower than those obtained with the same formulations at pH 6.8: the swelling percentage of F3 and F4 matrix tablets was 97.9 ± 2.9% and 95.8 ± 3.2% in the first hour, reaching a 188.0 ± 4.3% and 229.1 ± 0.8% swelling percentage after 8 h. The swelling process at this pH took place rapidly when the medium moistened the polymer matrix.

After 1 h in contact with FaSSIF, the dry core of the tablets was surrounded by a whitish layer of high viscosity gel. By increasing the contact time, the volume of the dry core decreased, and was transformed into gel, in such a way that after 8 h the dry core was negligible. Around the whitish layer of gel, a layer of transparent gel grows, being clearly distinguished in the photos after 8 h.

## 4. Discussion

Acrylic acid-based hydrogels show good physical characteristics for the elaboration of matrix tablets for extended release of drugs. In the present study, we evaluated the potential of a new poly(magnesium acrylate) hydrogel, called PAMgA, as a new excipient for extended released tablets using metformin HCl as a model drug. Metformin is a small molecule (MW = 129.16) that is classified as highly water-soluble and very rapidly dissolving drug, with all the dose dissolved in less than 15 min at pH 1.2; 4.5 (data not shown) and 6.8 (Figure 3-small graphics) [25]. Both characteristics make especially difficult to achieve the extended release of this drug from hydrophilic matrices.

The synthesis of PAMgA polymer was carried out in two stages, elaboration of the monomer and subsequently the polymerization stage. Since hydrogel characteristics are related to their polymeric network, PAMgA with two crosslinking degrees was synthetized: PAMgA 5 and PAMgA 40. The yield of the reaction for PAMgA 5 and PAMgA 40 was high, although slightly higher in the case of PAMgA 40. This difference could be attributed to the distillation process, as in the case of PAMgA 5 is more likely to disentangle the acrylic chains because of its lower the crosslinking degree.

The FT-IR spectra obtained with both hydrogels overlap, as the same behaviour occurs. With regard to DSC analysis, PAMgA 5 and PAMgA 40 showed no differences in the thermograms, so the crosslinking degree of the polymer is not a key factor in the thermal behaviour of the hydrogel. In a study on Carbopol^®^ polymers, Gómez-Carracedo et al. also found that their thermal behaviour was independent of their cross-linking degree and molecular weight [26]. In this study all Carbopol^®^ compacts showed a glass temperature between 130 and 140 °C. However, in our study, no thermal events were detected between −90 and 250 °C, which ensured that the polymer state is kept unaltered during tablet preparation and residence in the gastrointestinal tract following oral administration. Hetper et al. investigated the decomposition temperature of metal polyacrylates with other temperature range (25–600 °C). These authors indicated that the maximum decomposition temperature for magnesium polyacrylates was 460 °C and the polymer decomposed into magnesium oxide [27].

In a previous study [16], in vitro and in vivo toxicity of the polymer was evaluated. Cytotoxicity of the hydrogels was studied by a direct contact method and two indirect techniques (MTT colorimetric assay and flow cytrometry analysis). Cytotoxicity was tested on mouse embryonic fibroblast NIH-3T3 cells. In no case was cell damage evidenced. Toxicity in vivo was tested in mice by different routes (namely, intraperitoneal, subcutaneous and oral) at a dose ranging from 10 to 500 mg/kg. In no case was any physiological neither histological alteration evidenced, nor did the animals develop any symptoms. However, high doses by the oral route were not evaluated. In the current study, the oral biocompatibility studies in mice revealed that with doses of up to 10 g/kg no signs of toxicity were observed, changes in body weight and food consumption were not detected and symptoms as restlessness, squeaks, lack or hypermobility or vomiting were not reported. Diarrhoea was observed but only in some animals belonging to the highest dose group. The diarrhoea could be attributed to an osmotic effect of the polymer, present in high amounts in the faeces, rather than to a chemical irritation of the intestine caused by the polymer. The morphological analysis of the liver, spleen, lungs and kidneys after animal euthanasia did not show any alteration, even stomach and intestine showed an intact mucosa with a normal organ appearance. Sarkar et al. evaluated the toxicity of a poly(acrylic acid) grafted gellan gum (PAAc-g-GG) mucoadhesive polymer [13]. They reported that when LD50 is greater than 2 g/kg, the substance is considered as nontoxic. In our study, the maximum dose administrated was 10 g/kg of the polymer, clearly higher to that administered by Sarkar et al., and none of the animals died during the study. All these results suggest that PAMgA could be classified as a nontoxic excipient for oral dosage forms, and the maximum oral daily intake of PAMgA polymer could be at least 10 g/kg regardless of the crosslinking degree of the polymer.

Matrix tablets of PAMgA 5 and PAMgA 40 containing a dose of 500 mg of metformin HCl were prepared. Due to the high relative percentage of metformin within the formulation (a consequence of its high therapeutic dose), tables obtained by direct compression of the drug-polymer blend (F1 and F2) showed low mechanical resistance and capping tendency. Therefore, metformin was previously granulated with NaCMC as binder to obtain F3 and F4 matrix tablets, which facilitated the compression of the blend with the polymer and improved the mechanical resistance of the tablets.

Extended release of metformin was achieved from the four formulations of matrix tablets prepared, with 85% of the dose released between 6 and more than 10 h, depending on the formulation and the release media.

The drug release was seemingly influenced by the pH of the media, being slightly faster in FaSSGF than in FaSSIF (Figure 3). Biorelevant media were used in the in vitro drug release assays to better simulate physiological conditions. For the FaSSIF, the presence of bile salts (sodium taurocholate) and phospholipids (lecithin) facilitates the wetting of some drugs and excipients, making easier the penetration of the dissolution medium through the pores of the matrix structure. This effect could accelerate drug dissolution, especially in the case of poorly water-soluble drugs, where their solubility could also increase by micelles formation [28]. However, in the case of a highly water-soluble drug, as it is the case of metformin, this effect is negligible, and the different drug release rate could be attributed to the different behaviour of the polymer in both release media. Hydrogel matrices in contact with aqueous media gradually imbibe water in their structure and swell, forming an increasingly high viscosity layer of gel whereby the drug diffuses. Later, at the interface between the gel layer and the surrounding medium, the polymer chains gradually disentangle from the interface by erosion, thus enhancing drug release. The swelling and erosion studies were carried out in 250 mL of the two biorelevant media, FaSSGF (pH 1.2) and FaSSIF (pH 6.8). These processes are pH-dependent in the case of crosslinked acrylic acid polymers as Carbopol^®^, where at low pH the gel layer has lower viscosity than at pH > 5, facilitating matrix erosion [29]. As shown in Figure 4, in the case of PAMgA, matrix erosion is slightly more rapid at pH 1.2 than at pH 6.8, whereas in FaSSIF polymer swelling is more intensive than in FaSSGF. When the polymer becomes in contact with FaSSGF, the COOH groups are not ionized, and a lower swelling ratio is observed. The low viscosity of the gel layer formed favoured the erosion of the matrix. Swelling of the polymer occurs at a pH above the pKa of the carboxyl group of the hydrogel [30].

At pH 6.8 the COOH groups ionized into negatively charge species so this resulted in electrostatic forces that forced the hydrogel to expand, and therefore to increase the swelling ratio [31]. A high viscosity gel layer was formed, that delayed matrix erosion. In the case of F3 and F4 formulations, the presence of NaCMC as part of the gel layer could also contribute to their swelling capacity.

The erosion and swelling capacity of the PAMgA tablets were related to the metformin release rate, being slower at pH 6.8 than in acidic medium. On the one hand, matrix erosion conducted to drug release, in such a way that more than 85% of the drug was released from all the formulations after 8 h at pH 1.2; and on the other hand, the drug needed to go through the gel layer to be released from the tablet and this path was thicker and more viscous in FaSSIF, which lead to lower percentages of metformin released after 8 h at pH 6.8. Although the different behaviour of the matrix according to the release medium could account for the slightly faster release of metformin in FaSSGF than in FaSSIF, the difference is not greater due to the high solubility of the drug in both aqueous mediums.

Difference and similarity factors are a statistical tool widely used to measure the similarity of in vitro drug release profiles. When drug release from matrix tablets prepared with metformin incorporated in different way was compared, significant differences were detected in the release rate, being slightly slower in formulations wherein the metformin was incorporated as a powder (F1 and F2) than when the drug was previously granulated with NaCMC (F3 and F4). In order to determine the main mechanism responsible for drug release from the matrix tablets, dissolution data were fitted to Korsmeyer–Peppas equation. There are several possible mechanisms involved in drug release from the polymeric matrix: (a) drug diffusion through the gel layer of the matrix, (b) erosion of the polymer and (c) a combination of both mechanisms: diffusion of the drug and erosion of the hydrogel [20]. The values of the diffusional exponent (n) for F1 and F2 formulations (Table 7) indicate that the mechanism of metformin release was a combination of diffusion of the drug through the hydrated gel layer and matrix erosion (anomalous transport). The drug was entrapped within the polymeric network in the dry tablets, and as the contact time with the release medium increased, the polymer matrix progressively imbibed water and began to swell, forming the gel layer. As reported by Ghori and Conway, highly soluble drugs like metformin, are released by diffusing though the gel layer matrices and this mechanism is considered the principal way of release [32]. However, F1 and F2 tablets, with a 50% *w*/*w* of drug directly mixed with the polymer, showed lower hardness and tendency to capping, since metformin HCl is difficult to compress directly. This lower hardness could contribute to drug release by matrix erosion.

On the contrary, the values of diffusional exponent (n) for F3 and F4 formulations indicate that the drug release was mainly controlled by its diffusion through the gel layer (Fickian diffusion). Formulations F3 and F4 also include NaCMC to granulate the metformin HCl and it contributes to stabilize the gel layer. The presence of NaCMC increased the robustness and resistance of the hydrated gel layer of the matrices [33].

Rubio-Retama et al. postulated that the presence of crosslinking molecules in the PAMgA polymer introduced structural inhomogeneities that could modify the macroscopic properties of the polymer and its application [15]. However, when drug release from matrix tablets prepared with PAMgA of different degree of crosslinking were compared (F1 vs. F2 and F3 vs. F4), no significant differences were detected in FaSSGF neither, more clearly, in FaSSIF (Table 6). When swelling behaviour was compared only significant differences between F3 and F4 at all the tested times were detected in the experiment carried out in FaSSGF, showing a greater swelling capacity the F4 tablets (Figure 4). However, in FaSSIF the greater swelling capacity of F4 tables was only observed after 10 h in contact with the release medium. The increase in swelling ratio observed for F4 tablets, prepared with PAMgA 40, may be due to the increase of hydrophilic hydroxyl groups allowing water absorption into the hydrogel, in such a way that this effect of the crosslinking degree of the polymer on its swelling capacity results more evident for low capacity of swelling (acid pH). These differences in swelling behaviour related to crosslinking degree of the matrices did not affect the rate of metformin release, probably due to its high water-solubility.

In the US Pharmacopoeia, 10 dissolution tests for Metformin Hydrochloride Extended-Release Tablets are described, where apparatus II at 100 rpm is used. In all these tests dissolution medium pH 6.8 is used. Formulation 3 meets tolerance limits established for 500 mg tablets in three of these tests (test 10, test 11 and test 14), that are 25–45% dissolved after 1 h; 50–70% dissolved after 3 h and not less than 85% dissolved after 10 h.

**Figure 5:**
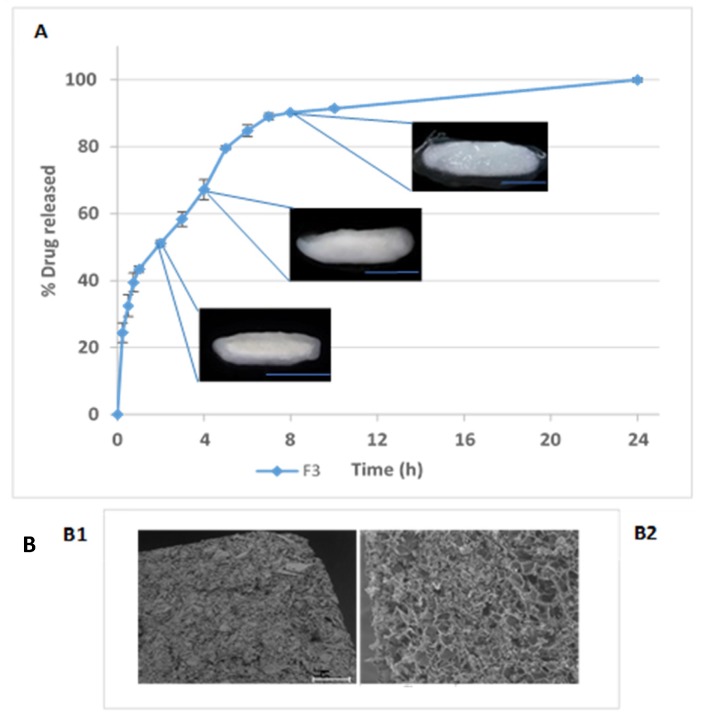
(**A**) F3 release profile and photograph of the tablets after 1, 4 and 8 h in FaSSIF (scale bar: 1 cm). (**B**) SEM images on tablet surface ((**B1**): dry tablets; (**B2**): tablets moistened following 8 h incubation with phosphate buffer and freeze-dried subsequently).

In Figure 5 metformin release from F3 tablets is represented together with photographs of a longitudinal cut of tablets along with the dissolution test. When the matrix tablets were moistened with the dissolution medium, they begun to swell and form the gel layer. The water penetrated the polymer chains from the outer side of the matrix until they reached the inner zone. Because of the relaxation of the polymer chains that leads to the formation of the gel layer, the tablets increased in size. The more water imbibed, the thicker the gel layer. Erosion process was a consequence of the disentanglement of the polymer chain in the external surface of the swelling tablets and led to a reduction in tablet size. The rate of drug release from hydrogel matrices is primarily governed by the rate and extent of polymer swelling, the dissolution of the drug in the absorbed water, the diffusion of the drug through the swelled polymer network and, finally, the erosion/dissolution rate of the swelled polymer chains. All these processes lead to the formation of three different fronts in a matrix structure: the swelling front, the diffusion front and the erosion front [34]. In the photos of Figure 5 these fronts can be differentiated:

- The swelling front (polymer glassy-rubbery region transition): this front was formed between the dry core of the tablet and the gel layer (also known as the rubbery region). This is clearly observed after 1 h in contact with the release medium; after 4 h it has moved inwards and after 8 h it is not possible to see any glassy inner core in any tablet.
- The diffusion front (solid drug-drug solution boundary): between the swelling and the erosion front. This front separates the zone where the drug is undissolved from the zone the drug has already dissolved in the gel layer and moves inwards. It is near the swelling front in matrices containing highly water-soluble drugs, so is thin and difficult to see [35]. This front cannot be seen in any photo due to the high water-solubility and very rapid dissolution of metformin HCl.
- The erosion front (swollen matrix-solvent): appears between the gel layer and the dissolution medium. This front disappears along the process of matrix erosion. This front is clearly visible in all the photos. After 8 h, a new front is clearly observed, delimiting high viscosity-low viscosity gel layers. At this point, the polymer was yet able to maintain the original shape of the tables after swelling.

## 5. Conclusions

PAMgA can be classified as a nontoxic excipient for solid oral dosage forms, with maximum oral daily intake of at least 10 g/kg regardless of the crosslinking degree of the polymer. This new hydrogel effectively controls the release of small and highly hydrophilic molecules when formulated in matrix tablets for oral administration, in such a way that metformin extended-release tables that meet USP specifications have been obtained. The rate of metformin release from PAMgA matrices is mainly controlled by its diffusion through the gel layer (Fickian diffusion). The swelling capacity and the erosion of the PAMgA tablets influence the metformin release rate, that is slower at pH 6.8, where polymer swelling is more intensive, than in gastric medium, where matrix erosion is slightly more rapid. The crosslinking degree of the polymer significantly influences its swelling capacity in acid pH, where swelling is moderate, but not in intestinal fluid, where swelling is more intensive.

## Figures and Tables

**Table 1:** Experimental groups for the oral biocompatibility assay.

Group	PAMgA 5	Group	PAMgA 40
I	1 ± 0.2 g/Kg	IV	1 ± 0.2 g/Kg
II	5 ± 1 g/Kg	V	5 ± 1 g/Kg
III	10 ± 2 g/Kg	VI	10 ± 2 g/Kg

**Table 2:** Composition of metformin tablets F1–F4.

Ingredients (g)	F1	F2	F3	F4
PAMgA 5	0.50	-	0.50	-
PAMgA 40	-	0.50	-	0.50
Metformin HCl	0.50	0.50	-	-
Metformin HCl granules	-	-	0.50	0.50

**Table 3:** Composition of the biorelevant media FaSSGF and FaSSIF.

Components	FaSSGF	FaSSIF
Sodium taurocholate	80 µM	3 mM
Lecithin	20 µM	0.75 mM
Sodium dihydrogen phosphate	-	3.438 g
Pepsin	0.1 mg/mL	-
Sodium chloride	34.2 mM	6.186 g
Hydrogen chloride qs ad	pH 1.2	-
Sodium hydroxide qs ad	-	pH 6.8
Deionized water	1 L	1 L
Osmolarity (mOsmol/Kg)	120.7 ± 2.5	~270
Surface tension (mN/m)	42.6	54
pH	1.2	6.8

**Table 4:** Clinical response after 24 h of oral administration of both PAMgA 5 and PAMgA 40 at doses between 1–10 g/kg.

Clinical Score	PAMgA 5 (g/Kg)			PAMgA 40 (g/Kg)		
Dose range	1 ± 0.2	5 ± 1	10 ± 2	1 ± 0.2	5 ± 1	10 ± 2
Restlessness	0	0	0	0	0	0
Squeaks	0	0	0	0	0	0
Mobility	0	0	0	0	0	0
Vomiting	0	0	0	0	0	0
Diarrhoea	0	0	1	0	0	1
Death	0	0	0	0	0	0

**Table 5:** Characterization of the different batches of PAMgA matrix tablets.

Tablet Characteristics	F1	F2	F3	F4
Weight (mg)	985.0 ± 5.4	989.0 ± 3.6	984.6 ± 6.6	988.5 ± 4.5
Thickness (mm)	6.46 ± 0.01	6.45 ± 0.01	6.46 ± 0.01	6.44 ± 0.01
Diameter (mm)	7.88 ± 0.01	7.89 ± 0.01	7.88 ± 0.01	7.87 ± 0.01
Length (mm)	14.05 ± 0.01	14.03 ± 0.01	14.04 ± 0.01	14.05 ± 0.01
Hardness (N)	64.5 ± 2.0	65.1 ± 1.5	134.7 ± 1.9	136.2 ± 2.3

**Table 6:** Values of *f*_1_ and *f*_2_ obtained in the comparison of the 4 formulations of tablets (F1, F2, F3, F4) in both pH assayed. S: similar; NS: non-similar.

	FaSSGF				FaSSIF			
	F1–F2	F3–F4	F1–F3	F2–F4	F1–F2	F3–F4	F1–F3	F2–F4
*f*_1_	17.2	2.8	15.8	32.2	4.2	0.4	39.8	42.5
*f*_2_	51.0	81.3	49.1	33.3	80.5	92.7	26.8	23.8
	NS/S	S/S	NS/NS	NS/NS	S/S	S/S	NS/NS	NS/NS

**Table 7:** Korsmeyer-Peppas model fitting of formulations F1–F4 of metformin HCl in FaSSGF and FaSSIF.

	FaSSGF				FaSSIF			
	Release Exponent (n)	Release Rate Constant (k)	Correlation Coefficient (r^2^)	Order Release	Release Exponent (n)	Release Rate Constant (k)	Correlation Coefficient (r^2^)	Order Release
F1	0.4917	37.273	0.9968	Anomalous transport	0.4924	26.564	0.9975	Anomalous transport
F2	0.5425	28.009	0.9973	Anomalous transport	0.4944	27.841	0.9968	Anomalous transport
F3	0.3772	51.404	0.9815	Fickian diffusion	0.3441	41.314	0.9906	Fickian diffusion
F4	0.3759	52.393	0.9825	Fickian diffusion	0.3117	42.276	0.9873	Fickian diffusion
